# Fluxes of lactate into, from, and among gap junction-coupled astrocytes and their interaction with noradrenaline

**DOI:** 10.3389/fnins.2014.00261

**Published:** 2014-09-09

**Authors:** Leif Hertz, Marie E. Gibbs, Gerald A. Dienel

**Affiliations:** ^1^Laboratory of Brain Metabolic Diseases, Institute of Metabolic Disease Research and Drug Development, China Medical UniversityShenyang, China; ^2^Drug Discovery Biology, Monash Institute of Pharmaceutical Sciences, Monash UniversityClayton, VIC, Australia; ^3^Department of Neurology, University of Arkansas for Medical SciencesLittle Rock, AR, USA

**Keywords:** astrocyte, acetate, lactate, locus coeruleus, neuron, monocarboxylic acid transporter, memory

## Abstract

Lactate is a versatile metabolite with important roles in modulation of brain glucose utilization rate (CMR_glc_), diagnosis of brain-injured patients, redox- and receptor-mediated signaling, memory, and alteration of gene transcription. Neurons and astrocytes release and accumulate lactate using equilibrative monocarboxylate transporters that carry out net transmembrane transport of lactate only until intra- and extracellular levels reach equilibrium. Astrocytes have much faster lactate uptake than neurons and shuttle more lactate among gap junction-coupled astrocytes than to nearby neurons. Lactate diffusion within syncytia can provide precursors for oxidative metabolism and glutamate synthesis and facilitate its release from endfeet to perivascular space to stimulate blood flow. Lactate efflux from brain during activation underlies the large underestimation of CMR_glc_ with labeled glucose and fall in CMR_O2_/CMR_glc_ ratio. Receptor-mediated effects of lactate on locus coeruleus neurons include noradrenaline release in cerebral cortex and c-AMP-mediated stimulation of astrocytic gap junctional coupling, thereby enhancing its dispersal and release from brain. Lactate transport is essential for its multifunctional roles.

## Metabolic, diagnostic, and signaling roles of lactate

Lactate has well-known and intriguing roles in brain function. Its resting concentration (~0.5–1 mmol/L) doubles during brain activation, and increases ~10–20-fold during abnormal states (Siesjö, 1978; Mangia et al., 2007). Lactate is generated from pyruvate when (i) glycolytic flux exceeds the rates of the TCA cycle and the malate-aspartate shuttle (MAS) that transfers reducing equivalents from cytoplasmic NADH into mitochondria, or (ii) when oxygen levels are insufficient to sustain oxidative metabolism. Thus, lactate formation is a “safety valve” to quickly regenerate NAD^+^ from NADH, thereby allowing rapid up-regulation and maintenance of high glycolytic flux. Lactate and pyruvate readily move down their concentration gradients to extracellular fluid, and the lactate/pyruvate concentration ratio in microdialysate is an important diagnostic tool predictive of clinical outcome of patients with traumatic brain injury; the higher the ratio the worse outcome (Nordström et al., 2013). Increased lactate production to sustain high glycolytic rate is associated with greater lactate release to blood because the brain concentration then exceeds that in blood. High cerebral blood flow maintains this gradient and “pulls” lactate from brain. Lactate in perivascular fluid, presumably mainly released from astrocytic endfeet (Gandhi et al., 2009), stimulates blood flow to activated regions (Laptook et al., 1988; Hein et al., 2006; Lombard, 2006; Yamanishi et al., 2006; Gordon et al., 2008), increasing nutrient delivery and by-product removal.

Conversely, increasing blood lactate concentration by intense physical activity drives lactate down its concentration gradient into all brain cells. Lactate oxidation supplements brain glucose metabolism to an increasing extent with rising blood level (Dalsgaard et al., 2004; Van Hall et al., 2009), and it does not accumulate in brain above resting levels (Dalsgaard et al., 2004). Metabolism of lactate requires its conversion back to pyruvate that, in turn, can have different metabolic fates (conversion to alanine, oxaloacetate, or acetyl CoA), which vary with cell type and metabolic state. Continued net uptake of lactate depends on its oxidation to pyruvate plus NADH and may cause the intracellular redox state to become more reduced, although cytosolic NAD^+^/NADH ratio is relatively stable in cell lines (Sun et al., 2012). Lactate is co-transported with a proton via equilibrative monocarboxylic acid transporters (MCTs) (Poole and Halestrap, 1993), and lactate influx accordingly causes intracellular acidification (Nedergaard and Goldman, 1993). Lactate uptake can, therefore, inhibit glycolysis by reducing availability of NAD^+^ for glycolysis and by acidification that can inhibit phosphofructokinase, which has a steep pH-activity profile (Dienel, 2012). Widespread lactate signaling, especially to neurons, via the receptor GPR81 decreases cAMP (IC_50_~29 mmol/L), which can decrease glycolysis at high extracellular lactate concentrations; a significant effect on cAMP requires ≥10 mmol/L lactate (Lauritzen et al., 2013). Thus, “pushing” lactate into all brain cells from blood provides supplementary fuel and evokes regulatory mechanisms that reduce brain glucose utilization when muscular lactate production is high.

Lactate can also influence astrocytic and neuronal activities by redox-mediated signaling. Astrocyte calcium signals are regulated by NAD^+^/NADH redox state (Requardt et al., 2012; Wilhelm and Hirrlinger, 2012), and changes in intracellular NAD^+^ and NADH levels arising from lactate fluxes may affect their binding to transcription factors and influence gene expression (Nakamura et al., 2012). For example, the transcription co-repressor, C-terminal binding protein (CtBP), is a dehydrogenase that undergoes conformational change with binding of NAD^+^ and NADH; NADH has a much higher affinity for CtBP, allowing it to serve as a redox sensor that destabilizes interactions with CtBP and transcription factors (Kumar et al., 2002; Fjeld et al., 2003). Increased NADH levels are thought to underlie seizure-induced increased expression of brain-derived neurotrophic factor (BDNF) and its receptor TrkB (Garriga-Canut et al., 2006). NAD^+^ is required for the action of sirtuins, a family of deacetylases that regulate activities of transcription factors and metabolic cofactors, and important roles for sirtuins in brain development, aging, and neurodegenerative diseases have been identified (Harting and Knoll, 2010; Bonda et al., 2011).

To summarize, lactate serves vital functions that include metabolic regulation (sustaining glycolysis by regenerating NAD^+^ or inhibiting glycolysis by intracellular acidification, NAD^+^ depletion and signaling), blood flow stimulation, influence on gene transcription via redox state, and signaling via receptor binding. During intense exercise muscle-derived lactate serves as an important metabolite for brain. Movement of lactate to and from cells via MCTs seems to be a central element in its multifunctional roles.

## MCT transporter function

Lactate is bi-directionally transported across cell membranes by MCT-mediated diffusional, saturable co-transport with H^+^. In the absence of a transcellular H^+^ gradient, extracellular lactate can increase its intracellular concentration up to, but not beyond the extracellular level and vice versa (Poole and Halestrap, 1993; Juel and Halestrap, 1999). Transporter-mediated diffusional uptake is equilibrative and energy-independent. However, continuing inwardly-directed diffusional *net* transport (influx) can be achieved by intracellular metabolism that reduces the intracellular level of the non-metabolized lactate and maintains a concentration gradient between extra- and intracellular concentrations of the non-metabolized compound (metabolism-driven uptake). This cannot increase the intracellular concentration of lactate itself. Analogously, continued removal of extracellular lactate by diffusion or uptake into other cells can increase net outward transport of lactate (efflux), but not its extracellular concentration. If extra- and intracellular pH differ, the equilibrium level is determined by the gradients of both lactate anions and H^+^, and it is reached when the product of intracellular lactate and H^+^ concentrations equals that of extracellular lactate and H^+^ concentrations. Extracellular pH in brain is normally 7.3, but it is lower in brain slices (~7.1) incubated at pH 7.3–7.4 (Chesler, 2003). Most results for intracellular pH have been obtained in brain slices or cultured cells and it is generally lower than in extracellular fluid although only by 0.2–0.3 pH units, indicating that the H^+^ concentration is at most two-fold higher intracellularly than extracellularly (e.g., Roos and Boron, 1981). Thus, the H^+^ gradient only moderately enhances diffusional lactate efflux and reduces its diffusional influx.

**Figure 1:**
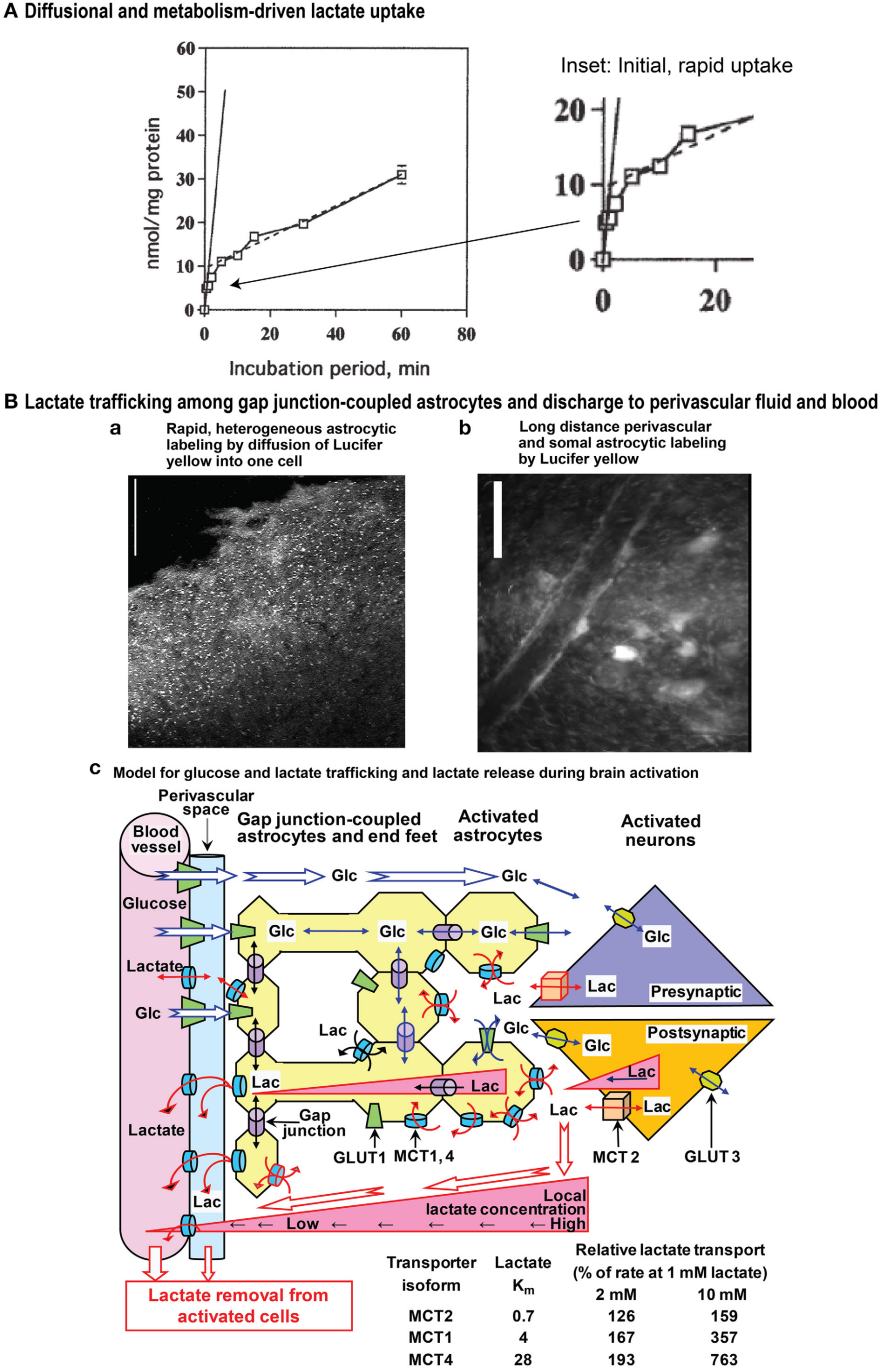
**Influx and gap junction-mediated trafficking of lactate**. **(A)** Diffusional and metabolism-driven lactate uptake. Accumulation of [U-^14^C]lactate into primary cultures of cerebellar granule cell neurons in primary cultures incubated in tissue culture medium of approximately similar pH as the intracellular water phase, shown as a function of time of exposure to 1 mmol/L [U-^14^C]lactate. The solid line is an extrapolation of the initial, rapid uptake by facilitated diffusion during the first few seconds at ~10 nmol/mg protein. The inset (right panel) emphasizes the early component of lactate uptake. The continued slower uptake of label after the initial rapid phase represents metabolism-driven uptake, and its rate, indicated by the stippled line, is sustained for at least an hour at 0.5 nmol lactate/mg protein per min. Slightly modified from Dienel and Hertz (2001), ©2001 Wiley-Liss, Inc., with permission of John Wiley and Sons, Inc. (**B)** Lactate trafficking among astrocytes. Gap junction-coupled astrocytes in slices of adult rat brain inferior colliculus were visualized by 5 min diffusion of Lucifer yellow from a micropipette inserted into a single astrocyte. Lucifer yellow labeled the soma (light spots) of as many as 10,000 astrocytes located up to about 1 mm from the impaled cell **(a)**, and diffusion of dye into astrocytic endfeet surrounding blood vessels caused high perivasculature labeling **(b)**. Scale bars in a and b denote 100 and 25 μm, respectively. Note that Lucifer yellow is retained within the coupled cells and it reveals the size of the syncytium coupled to a single astrocyte. Lactate can enter and leave cells via MCT transporters, and its direct diffusion (i.e., without exit and re-entry) throughout the extent of the entire Lucifer yellow-labeled syncytium is probably less than that of Lucifer yellow. Lactate was directly shown to diffuse through gap junctions to coupled cells located ~50 μm from the impaled cell (longer distances were not tested; Gandhi et al., 2009). Lactate exit plus re-entry into the same syncytium or to separate nearby syncytia would lead to extensive diffusion of lactate from the point source of the impaled cell. The schematic model for metabolite trafficking **(c)** illustrates uptake of glucose from blood into interstitial fluid and astrocytic endfeet, followed by diffusion of glucose down its concentration gradient from blood through extracellular fluid and the astrocytic syncytium, ultimately to the cells that are actively metabolizing glucose and creating a local sink toward which unmetabolized glucose diffuses. Detailed studies of (i) rates and capacities for lactate uptake from extracellular fluid into astrocytes and neurons and (ii) shuttling of lactate among gap junction-coupled astrocytes (yellow) compared with shuttling from astrocytes to neurons revealed that astrocytes have faster and greater capacity for lactate uptake and for lactate shuttling within the syncytium compared with neuronal uptake and transfer of lactate to neurons; glucose can also diffuse from an impaled astrocyte to neurons (Gandhi et al., 2009). Thus, astrocytic lactate uptake from interstitial fluid prevails, regardless of the cellular origin of the lactate. Once inside the syncytium (yellow) diffusion of lactate down its concentration gradient through gap junctions (purple cylinders) to other coupled astrocytes and their endfeet facilitates lactate discharge to perivascular fluid (blue) where it can be removed from brain by perivascular-lymphatic flow and by discharge into cerebral venous blood. The perivascular fluid space is color coded only to emphasize its location; there is no physical boundary between interstitial fluid and perivascular fluid, although diffusion between these locations is influenced by tortuosity. Isoforms of monocarboxylic acid transporters (MCTs) have different K_m_ values for lactate, and relative rates of lactate transport by these isoforms when lactate concentration rises are illustrated in the table for K_m_ values within the ranges given in the text (i.e., 0.7, 3–5, and 15–30 mmol/L for MCT2, 1, and 4, respectively). The low K_m_ MCT2 in neurons restricts lactate influx and efflux compared with the higher Km isoforms in astrocytes. During brain activation in sedentary subjects, brain lactate level in activated structures is higher than that in blood. Triangles denote outward lactate gradients from intracellular to extracellular fluid, from extracellular fluid to blood, and from intracellular fluid of astrocytes located near cells with high glycolytic activity to endfeet and blood. During strenuous physical exercise that greatly increases blood lactate concentration, these gradients would be reversed, driving lactate into all brain cells (not shown). Glc, glucose; Lac, lactate; GLUT, glucose transporter. Modified from Gandhi et al. (2009) ©2009, the authors. Journal compilation ©2009 International Society for Neurochemistry, with permission from John Wiley and Sons, Inc and the authors.

Diffusional uptake is only measurable during very short incubation times and contribution of metabolism-driven uptake will distort its kinetics (Hertz and Dienel, 2005). Figure 1A illustrates lactate uptake into cerebellar neurons at 1 mmol/L extracellular lactate. The initial diffusional uptake is very brief (<~30 s; Figure 1A inset), rapid (~10 nmol/mg protein or 1 μmol/g wet wt.), and only occurs in cells containing <1 mmol/L lactate. Thereafter, metabolism-driven net uptake takes over and is sustained for ≥1 h at 0.5 nmol lactate/mg protein per min, corresponding to 0.25 nmol glucose equivalent/mg protein per min. Lactate metabolism is lower than measured rates of non-stimulated and stimulated glucose oxidation (1.0 and 2.23 nmol/mg protein per min, respectively) in cerebellar neurons (Peng et al., 1994). The above glucose oxidation rates are minimal values because the assays were based on ^14^CO_2_ production, and exchange reactions cause label dilution in amino acid pools, slowing ^14^CO_2_ release and causing underestimation of oxidation rate. Thus, the *potential* contribution of any lactate to total CO_2_ formation in the neurons under activated conditions would be <10% of that from glucose. In cultured astrocytes, diffusional uptake is faster than in neurons (suggesting higher V_max_), but the rate of metabolism-driven uptake is similar (Dienel and Hertz, 2001).

Neurons and astrocytes express different MCTs. MCT2 has a K_m_ for lactate of ~0.7 mmol/L and is predominantly neuronal, whereas MCT1 (K_m_ 3–5 mmol/L) and MCT4 (K_m_ 15–30 mmol/L) are mainly astrocytic (for references see Hertz and Dienel, 2005). These MCT's do not determine net lactate fluxes, which are mainly metabolism-driven for influx or concentration gradient-driven for efflux (although potentially increased by lactate removal by extracellular diffusion or cellular re-uptake), but they may be rate-limiting when concentration gradients develop rapidly. Lactate transport is governed by lactate concentration, K_m_, and transporter number, and it is enhanced by “transacceleration” (Juel, 1991; Juel et al., 1994). Lactate exit is stimulated by extracellular pyruvate (San Martin et al., 2013), perhaps stimulating a heteroexchange. The lower affinity MCTs in astrocytes may promote astrocytic release and re-uptake even at high concentrations. MCTs are inhibited by several drugs, including 4-CIN, and lactate transport is competitively inhibited by D-lactate. These toxins have repeatedly been used to allegedly show the importance of MCT-mediated intercellular transport. However, it has never been demonstrated that these drugs at the same concentrations do not also inhibit pyruvate uptake into mitochondria, as shown by McKenna et al. (2001), who demonstrated that incubation with 0.25 mmol/L 4-CIN decreased oxidation of glucose to ~50% of control values in both astrocytes and neurons in primary cultures, although cellular glucose uptake was not inhibited by 4-CIN.

Acetate is a preferential substrate for astrocytic, but not neuronal, MCTs, and it is also metabolized by astrocytes (Muir et al., 1986; Waniewski and Martin, 1998). Acetate may, accordingly, serve as an indicator of astrocyte-specific lactate transport. Inhibition of learning in day-old chicks by the non-metabolizable D-lactate can be prevented by administration of acetate at two different time periods, immediately after training and 20 min later (Gibbs and Hertz, 2008). Immediately after training, rescue by acetate requires co-administration of aspartate, which alone has no effect. Twenty min after training acetate by itself rescues learning; this is a time at which astrocytic metabolism is known to be activated, a further indication that acetate rescues energy metabolism. These observations identify the affected cells as astrocytes, and the aspartate requirement shows that the rescue immediately after training is due to formation of glutamate, which is normally formed in astrocytes from lactate/pyruvate by a combination of pyruvate carboxylation to oxaloacetate (which is astrocyte-specific) and pyruvate metabolism via the pyruvate dehydrogenase. No pyruvate carboxylation is possible with acetate as sole substrate, but co-administration of aspartate abolishes this requirement, because aspartate is an alternative oxaloacetate precursor. Thus, at both times, the rescue by acetate is due to support of *astrocytic* metabolism impaired by D-lactate, not to MCT-mediated inhibition of *neuronal* lactate uptake.

## Brain lactate fluxes

Because lactate transport is concentration-gradient driven, knowledge of both transport and metabolism is needed to evaluate net fluxes and ultimate fate of transported lactate. Microdialysis and microelectrode studies have shown that extracellular lactate levels rise quickly to about twice the resting value of ~0.5–1 mmol/L during an activating stimulus, then return to normal; up-and-down cycling of extracellular and total lactate concentrations occurs with repeated transient stimuli (e.g., Korf and De Boer, 1990; Mangia et al., 2007). Changes in lactate concentration reflect net input and output fluxes to the lactate pool and are not indicators of lactate *flux through* the lactate pools. Most extracellular lactate produced during brain activation may come from astrocytes (Elekes et al., 1996), but modeling supports a neuronal origin and shuttling to astrocytes (Mangia et al., 2009).

Small amounts of lactate, equivalent to ~5% of the glucose entering brain, are released to blood under resting conditions (Quistorff et al., 2008; Dienel, 2012), whereas during activation considerable quantities of lactate are released from brain to blood, both directly (22% during spreading depression; Cruz et al., 1999) and via the perivascular-lymphatic drainage system (Ball et al., 2010). Lactate efflux causes (i) a large (~50%) underestimation of the calculated rate of glucose utilization (CMR_glc_) when assayed with labeled glucose, in contrast to labeled deoxyglucose that is quantitatively trapped after its initial phosphorylation and (ii) a fall in the CMR_O2_/CMR_glc_ ratio due to greater rise in glucose utilization than oxygen consumption (Dienel, 2012). These two events reflect lactate release and occur under various conditions, e.g., sensory stimulation (Fox et al., 1988) and mental testing (Madsen et al., 1995) of humans and spreading depression (Adachi et al., 1995; Cruz et al., 1999) and sensory stimulation (Madsen et al., 1999; Schmalbruch et al., 2002) of rats. The CMR_O2_/CMR_glc_ ratio also falls with increased lactate uptake into brain during vigorous exercise (Quistorff et al., 2008). A common factor in all these situations may be an increase in extracellular lactate concentration.

## Cellular lactate uptake shuttling

To compare astrocytic and neuronal rates and capacities for uptake of lactate from extracellular fluid and for its transcellular shuttling, Gandhi et al. (2009) devised a real-time, selective, sensitive assay to measure lactate concentration in single cells in adult rat brain slices. At 2 mmol/L extracellular lactate, the approximate concentration during brain activation, initial rates of lactate uptake into astrocytes were twice those of neurons, and over the range 2–40 mmol/L the initial rate of diffusional lactate uptake into astrocytes was four-fold greater than that into neurons. The capacity for lactate uptake into astrocytes was also double that of neurons over this range. Because as many as ten thousand astrocytes are coupled via gap junctions (Ball et al., 2007) (Figures 1Ba,b), lactate can diffuse down its concentration gradient to other astrocytes within the large syncytium, as shown directly for coupled cells located ~50 μm apart (Gandhi et al., 2009). The initial rate of transfer among coupled astrocytes increased with lactate concentration from 0 to 5 mmol/L, whereas there was no concentration dependence of lactate transfer to neurons; net lactate transfer to another astrocyte was about five-fold greater than transfer to an equidistant neuron.

Together, these findings demonstrate that astrocytes avidly take up extracellular lactate, and quickly distribute the lactate to other astrocytes within the syncytium. There is a small, slower uptake of extracellular lactate by neurons and low transfer rate from astrocytes to neurons. Astrocytic endfeet surround capillaries and are also connected together via gap junctions (Figure 1Bb). Some of the lactate diffuses via its concentration gradient within the syncytium to endfeet where it can be released to perivascular fluid and ultimately to cerebral venous blood (Figure 1Bc) (Gandhi et al., 2009; Dienel, 2012), where it can stimulate blood flow that also washes out lactate from perivascular space fluid. Because more glucose is delivered to brain than is phosphorylated, release of a portion of excess fuel as lactate is not an energetic waste when viewed from a whole-body perspective. Other organs oxidize the released lactate.

## Influence of noradrenaline on lactate trafficking

The reduced CMR_O2_/CMR_glc_ ratio during activation is prevented by propranolol, an inhibitor of β-adrenergic signaling. In control rats, the CMR_O2_/CMR_glc_ ratio fell from 6.1 to 4.0 after stimulation of brain activity by release from their shelter boxes, and it rose back to 5.8 after the animals re-entered the box. After propranolol administration, the CMR_O2_/CMR_glc_ ratio remained unaltered during rest, stimulation, and recovery (6.2, 6.3, 6.4) (Schmalbruch et al., 2002). Thus, (i) stimulation activates glycolysis in stimulated region(s) with much less effect on oxidative metabolism, (ii) this effect is dependent on β-adrenergic stimulation, and (iii) there must be efflux of a glucose metabolite, e.g., lactate, from the stimulated area. Part of the reduction in CMR_O2_/CMR_glc_ ratio during brain activation may also reflect retention of some glucose in tissue by (i) an increase in lactate, (ii) use of glucose for glycogen synthesis, and (iii) increased pyruvate carboxylation (Öz et al., 2004) leading to enhanced glutamate formation (Gibbs et al., 2007; Mangia et al., 2012). The reduced CMR_O2_/CMR_glc_ ratio during exercise is also inhibited by propranolol (Quistorff et al., 2008; Gam et al., 2009).

Inhibition by propranolol of an activation-induced fall in CMR_O2_/CMR_glc_ ratio is consistent with a recent demonstration that specifically locus coeruleus (LC) neurons (the principal source of noradrenaline to brain cortex (Moore and Bloom, 1979), including astrocytes (Bekar et al., 2008), are stimulated by L-lactate, independent of its caloric value (Tang et al., 2014). Release of L-lactate from cultured astrocytes excites LC neurons and triggers release of noradrenaline, and physiologically-relevant concentrations of exogenous L-lactate (EC_50_ ~0.5 mmol/L) mimics these effects (Tang et al., 2014). The effects of L-lactate were stereo-selective, independent of its uptake into neurons, and involved a cAMP-mediated step. *In vivo* injections of L-lactate in the LC evoked arousal similar to the excitatory transmitter, L-glutamate. Because (i) lactate release is associated with activation-induced decreases in CMR_O2_/CMR_glc_ ratio (inhibited by propranolol) and (ii) astrocytic gap junction conductivity is up-regulated by cAMP, an intermediate in β-adrenergic signaling (Enkvist and McCarthy, 1994) blockade by propranolol may reduce gap junction-mediated lactate transport and release from brain.

**Figure 2:**
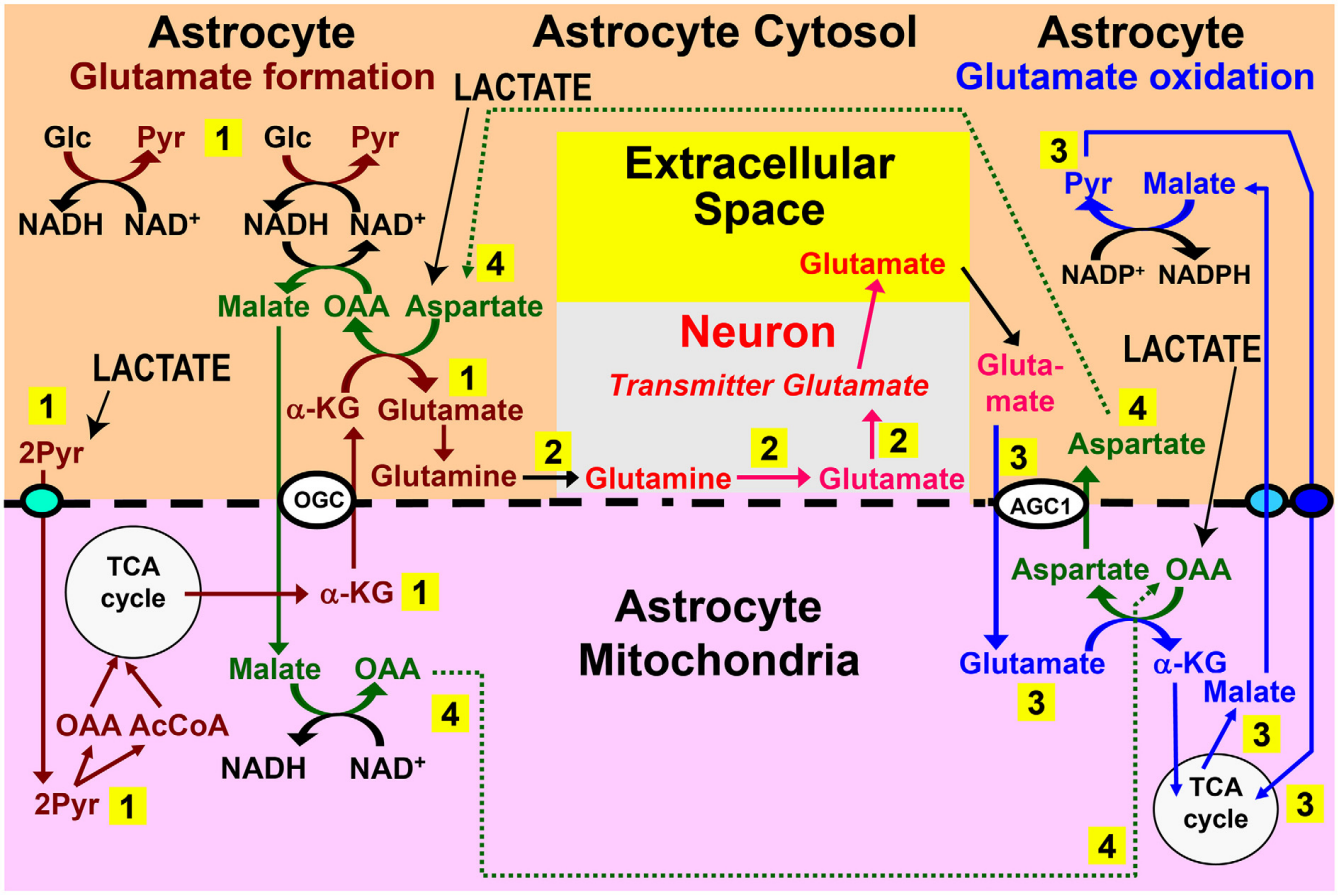
**Role for trans-astrocytic lactate trafficking in glutamate turnover**. Why would the brain want a lactate transport from one astrocyte to different neighboring astrocytes? One possibility is that lactate-pyruvate interconversions could be of importance for proposed pathways linking glutamate formation, which is astrocyte-specific, with its oxidative degradation, which may also be mainly or exclusively astrocytic (see papers cited in Hertz and Rodrigues, 2014). The proposed pathways linking glutamate synthesis, excitatory neurotransmission, and glutamate oxidation are illustrated in this figure. Pathway 1 (numbered in yellow rectangle) shows the proposed cytosolic-mitochondrial metabolite trafficking associated with astrocytic production of glutamine. Pathway 2 shows glutamine transfer from astrocytes to glutamatergic neurons and extracellular release of transmitter glutamate. Pathway 3 illustrates subsequent re-uptake of glutamate and its oxidative metabolism in astrocytes. Pathway 4 provides the necessary aspartate- and oxaloacetate-dependent connections between pathways 1 and 3, with all pathways located in the same cell. A major problem with this model is that glutamate formation and oxidation may not occur in the same astrocyte, but, instead, in spatially-separated astrocytes. Trans-astrocytic lactate transport and its subsequent conversion to pyruvate and carboxylation would allow rapid synthesis of oxaloacetate (OAA) and aspartate that are needed for oxidation and synthesis of glutamate, respectively, according to this model (pathway 4) (lower right corner for OAA and upper left corner for aspartate). Lactate influx (shown in capital letters and with black arrows) could compensate for a lack of trafficking of these two compounds (pathway 4) between *spatially separated* glutamate-synthesizing and glutamate-oxidizing astrocytes. In addition, provision of lactate-derived pyruvate to astrocytes would provide a faster source than glucose for provision of the precursor carbon skeleton, and if only one of the two glucose molecules is replaced with pyruvate, malate would still be able to enter the mitochondria during glutamate synthesis. Biosynthesis of glutamine is shown in brown, and metabolic degradation of glutamate in blue. Redox shuttling and astrocytic release of glutamine and uptake of glutamate are shown in black, and neuronal hydrolysis of glutamine to glutamate and its release is shown in red. Reactions involving or resulting from transamination between aspartate and oxaloacetate are shown in green. Lactate could provide pyruvate for many of the reactions in these pathways in many astrocytes. AGC1, aspartate-glutamate exchanger, aralar; α-KG, α-ketoglutarate; Glc, glucose; Pyr, pyruvate; OGC, malate/α-KG exchanger. Slightly modified from Hertz (2011), with permission of the author. ©2011 International Society for Cerebral Blood Flow and Metabolism, Inc.

There might be additional beneficial effects of an adrenergically-stimulated, gap junction-mediated astrocyte-to-astrocyte lactate trafficking. Subsequent conversion of lactate to pyruvate would boost synthesis of oxaloacetate since pyruvate carboxylation in liver (and probably also in astrocytes) is stimulated by α-adrenergic activity (Garrison and Borland, 1979). Oxaloacetate is rapidly converted to aspartate which causes a 50% increase of astrocytic glutamate production (Pardo et al., 2011), consistent with increased mitochondrial glutamate formation by aspartate addition (Von Korff et al., 1971). Based on this aspartate dependence of glutamate formation and consistent with rapid astrocytic oxidative degradation of glutamate (McKenna, 2013; Whitelaw and Robinson, 2013), an interaction between glutamate synthesis and degradation has been suggested (Hertz, 2011). This interaction, illustrated in Figure 2 and described in its legend, would make the aspartate formed during glutamate oxidation available during glutamate synthesis. Moreover, use of aspartate transaminase, rather than of glutamate dehydrogenase in the inter-conversion between α-ketoglutarate and glutamate is consistent with predominant transamination-dependent glutamate degradation in brain mitochondria (Balazs, 1965; Dennis et al., 1977) vs. extensive use of glutamate dehydrogenase by cultured, isolated astrocytes (Yu et al., 1982; McKenna et al., 1996). However, the Figure 2 schematic shows cycling of aspartate and oxaloacetate within one astrocyte, and a problem with this model is that glutamate synthesis and its subsequent oxidation may occur in different astrocytes. Lactate transport between synthesizing and degrading astrocytes could rectify this problem by providing a substrate for rapid synthesis of both oxaloacetate and aspartate in the cells receiving lactate (Figure 2, black arrows), which could also partly replace glucose in α-ketoglutarate/glutamate synthesis. The huge flux in this cycle (Sibson et al., 1998; Rothman et al., 2011) and high rates of glutamate neosynthesis, accounting for 15–30% of the flux are consistent with the major trans-astrocytic lactate fluxes indicated by the large difference between glucose oxidation and total glucose utilization rates determined with glucose and deoxyglucose, which was described above.

## Concluding remarks

Lactate transport between brain cells is mainly among astrocytes and occurs both via gap junctions and release to extracellular space. The latter mechanism is important for LC-adrenergic signaling, and it also leads to a significant exit of lactate from the brain via peri-capillary flux and the lymphatic system. Adrenergic signaling plays a role in regulating lactate fluxes, and inter-astrocytic lactate flux may assist glutamate production and degradation in the glutamate-glutamine cycle.

### Conflict of interest statement

The authors declare that the research was conducted in the absence of any commercial or financial relationships that could be construed as a potential conflict of interest.
